# The Interchangeability of Plasma and Whole Blood Metal Ion Measurement in the Monitoring of Metal on Metal Hips

**DOI:** 10.1155/2015/216785

**Published:** 2015-12-20

**Authors:** Ibrahim A. Malek, Joanne Rogers, Amanda Christina King, Juliet Clutton, Daniel Winson, Alun John

**Affiliations:** ^1^All Wales Orthopaedic Training Programme, Cardiff CF14 4XW, UK; ^2^University Hospital of Wales, Heath Park, Cardiff CF14 4XW, UK; ^3^North Yorkshire Orthopaedic Training Programme, Huddersfield HD3 3EA, UK

## Abstract

One hundred and twenty six paired samples of plasma and whole blood were measured with inductively coupled plasma mass spectrometry technique for metal ions analysis to determine a relationship between them. There was a significant difference between the mean plasma and whole blood concentrations of both cobalt (Co) and chromium (Cr) (*p* < 0.0001 for both Co and Cr). The mean ratio between plasma and whole blood Cr and Co was 1.56 (range: 0.39–3.85) and 1.54 (range: 0.64–18.26), respectively, but Bland and Altman analysis illustrated that this relationship was not universal throughout the range of concentrations. There was higher variability at high concentrations for both ions. We conclude that both these concentrations should not be used interchangeably and conversion factors are unreliable due to concentration dependent variability.

## 1. Introduction

There has been a resurgence of Metal on Metal (MoM) hip arthroplasty over the last two decades but some implants have had high early failure rates and there is widespread reporting of Adverse Reaction to Metal Debris (ARMD) following MoM hip arthroplasty [1–5]. The follow-up, screening, and treatment of patients with MoM bearings are a challenging problem.

The Medicines and Healthcare Products Regulatory Agency (MHRA), UK, issued the first MoM hip alert in April 2010 and subsequently advised against the use of Large Head MoM Total Hip Replacement (THR) implants [6]. The MHRA recommended regular follow-up of these patients and suggested that whole blood metal ions levels should be obtained from these patients for early identification of failing MoM implants [7].

A Large number of MoM THR procedures were carried out at our institution between 2003 and 2009. Measurement of Co and Cr levels was part of the assessment of MoM hip implants in our surveillance clinic which was established prior to the publication of the MHRA guidelines. We chose to use plasma samples for metal ions analysis which was already established [8]. The measurements are undertaken at a hospital laboratory overseen by Trace Elements External Quality Assessment Scheme (TEQAS), UK.

The MHRA guidelines on MoM implants are based on whole blood metal ion analysis. We wanted to investigate whether the plasma levels which we had measured were interchangeable with whole blood levels and whether conversion factors could be used between the two. There is conflicting evidence about interchangeable use of cobalt (Co) and chromium (Cr) levels in serum and whole blood [9, 10]. The aim of this study was to evaluate the relationship between Co and Cr levels in plasma (used at our laboratory) and whole blood (recommended by the MHRA and US Food and Drug Administration) and decide future metal ions analysis strategy in our region for the surveillance of patients with MoM bearings hips.

## 2. Patients and Methods

We gained Institutional Review Board approval for measurement of various fractions of blood in these patients. An informed consent was obtained from all the patients. The blood samples were collected from sequential patients without any exclusion criteria to reproduce the heterogeneity of a daily laboratory practice. The lead biochemical scientist was blinded to implant brand and size. We only included patients who had their primary procedure performed at out centre to ascertain accurate and quick access to patients' demographics, implantation time, implant brand, and size of the implant if required.

Ten mls of venous blood was taken using a 21-gauge needle connected to two sequential sodium heparin containing trace element vacutainers (Vacuette, Greiner Bio-One GmbH, Austria). The sample was sent immediately to the laboratory for analysis. The metal ions analysis was carried out by the Department of Biochemistry at our hospital, which is a participating laboratory in the Trace Elements External Quality Assessment Scheme (TEQAS) in the UK. One vacutainer was centrifuged within four hours of venepuncture and the plasma separated. These plasma samples were stored along with the whole blood sample at 4°C pending analysis. Both these samples were processed within 48 hours of sample collection. Whole blood and plasma samples were analysed separately as they were calibrated against matrix matched standards.

### 2.1. Cobalt and Chromium Analysis

Ten millilitres of venous blood was obtained using a 21-gauge needle connected to two sequential sodium heparin trace element vacutainers (Vacuette, Greiner Bio-One GmbH, Austria). One vacutainer was centrifuged within four hours of venepuncture and the plasma separated and stored along with the whole blood sample at 4°C pending analysis. Cobalt (Co) and chromium (Cr) were measured using an Agilent 7700x inductively coupled plasma mass spectrometer (Agilent Technologies, Berkshire, UK). Samples, standards, and quality control material were diluted 1 in 15 with diluent containing 0.01% triton (Romil, Cambridge, UK), 0.01% EDTA (AnalaR, VWR, Lutterworth, UK), 0.2% ammonia (Romil, Cambridge, UK), and 20 ppb Gallium as an internal standard (Inorganic Ventures, Madrid, Spain). Isotopes 59 and 52 were measured for Co and Cr, respectively, using helium gas for interference correction. Whole blood and plasma samples were analysed separately as they were calibrated against matrix matched standards. The lowest detection limits for Co and Cr were 0.06 *μ*g/L.

### 2.2. Statistical Tests

Wilcoxon rank sum test for nonparametric data was used to examine the relationship between plasma and whole blood Co and Cr. Spearman correlation was used to assess correlation between Co and Cr in both blood fractions. A *p* value of ≤0.05 was considered significant and confidence intervals were computed at the 95% confidence level. The agreement between plasma and whole blood Co and Cr was assessed using Bland and Altman limits of agreement which is a simple graphical technique to assess agreement between two methods of clinical measurement [11]. The upper and lower limits of agreement for Bland and Altman analysis were calculated as mean difference ±1.96 standard deviation, respectively. The coefficients, influential points, and correction factors were derived from regression analyses using standard methods. The adjusted Bland and Altman analyses were performed following application of these correction factors to assess interconvertibility between plasma and whole blood concentrations.

Statistical analysis was carried out using SPSS 19.0 software (IBM SPSS, Chicago, United States).

## 3. Results

Paired samples of whole blood and plasma were obtained from 126 patients. Six samples were below detection limit of 0.06 *μ*g/L and these were excluded from statistical analysis leaving 120 paired samples for final analysis.  Table 1 illustrates the patient and procedure demographics.

The mean plasma and whole blood results were 3.99 (0.11–25.08) and 3.18 (0.09–20.21) *μ*g/L for Co and 3.15 (0.41–27.23) and 1.88 (0.36–13.73) *μ*g/L for Cr, respectively. This difference was statistically significant (*Wilcoxon rank sum test*: *p* < 0.0001 for both Co and Cr).

There was significant correlation between Co and Cr in both plasma and whole blood (Spearman correlation: 0.757 for Cr and 0.763 for Co, *p* < 0.0001 for both). The Co levels had significant correlation between plasma and whole blood (Spearman correlation: *p* < 0.0001); similarly the Cr levels also had significant correlation between plasma and whole blood (Spearman correlation, *p* < 0.0001).

**Figure 1:**
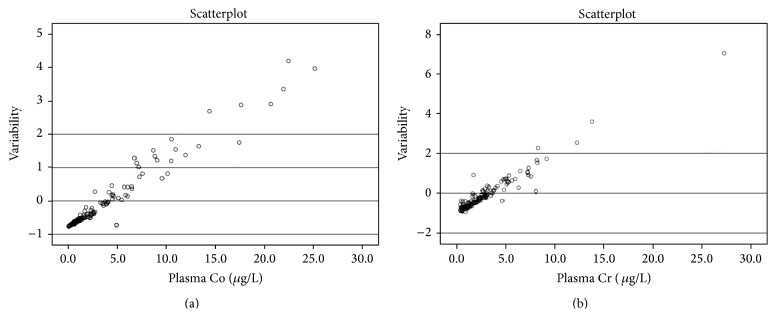
Scatterplot diagrams showing variability of concentration of Co (a) and Cr (b) in plasma per unit concentration in whole blood. The scatter demonstrates that the variability between plasma and whole blood is not uniform throughout the range of measurements and is concentration dependent.

Figure 1 demonstrates the scatterplot diagram of variability of concentration of Co and Cr in plasma per unit concentration in whole blood. At higher concentrations (i.e., on the right of the scale on the *x*-axis, blood concentrations tending towards 15 *μ*g/L) there is more variability, but at lower concentrations (left of the scale, blood concentrations tending towards 0 *μ*g/L) the variability is less.

The mean difference between serum and whole blood concentrations was 0.8045 *μ*g/L (−3 to 17.2) and 1.268 *μ*g/L (−4.4 to 22.7) for Co and Cr, respectively. The differences for both were statistically significant (*Wilcoxon rank sum test*, *p* < 0.0001). The normalised scatter (Figure 1) showed that the variability was greater in the higher range of concentrations compared with the lower ranges.

**Figure 2:**
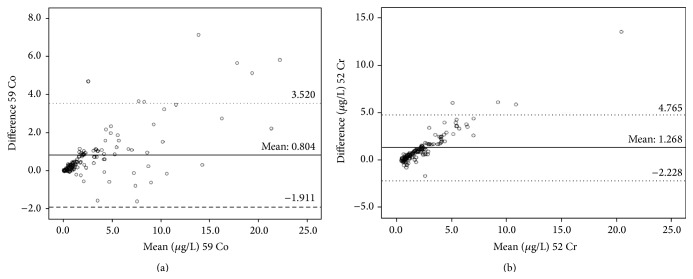
The scatterplot diagrams showing Bland and Altman limits of agreement between measurements in plasma and whole blood for Co (a) and Cr (b). Amongst these, seven cobalt data points and four chromium data points lie outside the range displayed but are included in the calculation.

**Figure 3:**
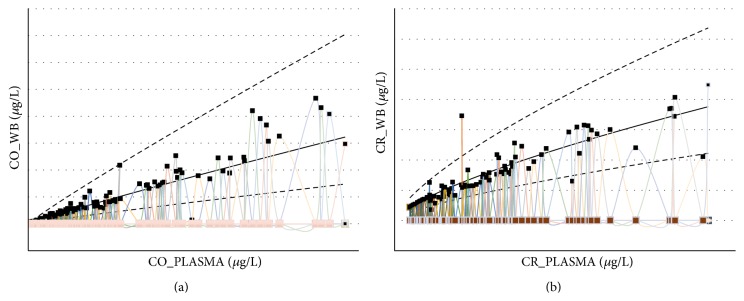
The scatterplot diagrams showing regression analysis of (a) cobalt and (b) chromium concentration in whole blood and plasma. The solid line represents the regression of whole blood on plasma. The broken lines represent the confidence limits for prediction. The potentially overinfluential observations have been removed.

Bland and Altman analyses (Figure 2) show the upper and lower limits of agreement (±1.96 SD) of 3.520 *μ*g/L to −1.911 *μ*g/L and 4.765 *μ*g/L to −2.228 *μ*g/L for Co and Cr, respectively. The difference plots of Bland and Altman (Figure 2) suggest that there is a trend; therefore a multivariate regression model was fitted to the data (Figure 3). For the Co data, a regression model was fitted giving coefficients of *α* = 0.214 (95% CI −0.051 to 0.479) and *β* = 1.186 (95% CI 1.134 to 1.237). After an analysis of the residuals, seven points were found to be influential. Following their removal, the coefficients became *α* = −0.394 (95% CI = −0.711 to −0.076) and *β* = 1.881 (95% CI = 1.707 to 2.054). Similarly, the model gave coefficients of *α* = −0.469 (95% CI, −0.707 to −0.231) and *β* = 1.922 (95% CI, 1.828 to 2.016) for Cr. An analysis of the residuals suggested 15 measurements were influential. If these are removed, the coefficients then become *α* = 0.195 (95% CI, −0.30 to 0.420) and *β* = 1.225 (95% CI, 1.174 to 1.276).

Multivariate regression was employed to model the whole blood ion concentration in terms of the plasma ion concentration for both cobalt and chromium. All the data was included and because both raw variables were skewed in each case, it was necessary to use the log of each in the model to correct for this. Other factors (patient age, time since surgery, gender, and type of surgery, unilateral or bilateral) were considered as confounders with only the type of surgery (unilateral versus bilateral) being significant (Wilcoxon rank sum test, *p* < 0.05 in both cases) and then being included in the model.

**Figure 4:**
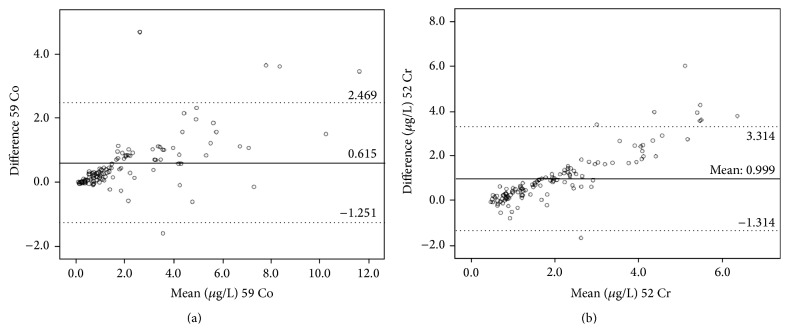
Applying the correction factors *β* = 1.881 cobalt and *β* = 1.225 chromium obtained from the adjusted regression analyses reduces the mean difference between serum and whole blood concentrations of cobalt (a) and chromium (b) to 0.615 (95% CI: −1.251 to 2.469) and 0.999 (95% CI: −1.314 to 3.314) and positions the limits of agreement more symmetrically on either side of zero.

In both cases the model fit was good (*R* ^2^ > 0.8) as shown in Figure 3. The actual data points are seen with the mean fit and confidence intervals from the model for the unilateral cases only. The width of the confidence intervals increases with plasma ion concentration as a consequence of the conversion back from the logs of the variables that were necessarily used in the model. This implies that the accuracy with which whole blood concentrations can be estimated from plasma concentrations lessens with increasing plasma concentration and therefore there may be a cut-off point before which it is reasonable to use plasma concentrations to estimate whole blood concentrations between upper and lower bounds. Multivariate regression analysis showed better agreement between whole blood and plasma for Co than Cr (*R* ^2^ = 0.9451 and 0.9325, resp.).  Figure 4 demonstrates the Bland and Altman analyses following removal of these influential points and application of correction factors for Co and Cr, respectively.

*“Concentration Paradox” Phenomenon of Conversion Ratios*. The mean ratio between plasma and whole blood Cr and Co was 1.56 (0.39 to 3.85) and 1.54 (0.64 to 18.26), respectively, derived from this data, although universal application of these constant conversion ratios is flawed. Figure 1 demonstrates that the variability is concentration dependent and application of these constant conversion ratios will show the concentration “paradox” phenomenon. The application of constant correction factors at lower plasma concentrations where the variability is low will render “overcorrection” for whole blood Co and Cr levels. Similarly, at higher plasma concentrations where the variability is high, it renders “undercorrection” for whole blood levels.

## 4. Discussion

After initial setback in 1960s, MoM hip articulation gained a significant second wave of popularity over the last two decades due to advances in implant manufacturing and engineering as well as a better understanding of MoM bearing tribology [1, 12–15]. It is inevitable that wear of the hip bearing takes place with activity and this leads to release and accumulation of metal ions in the body [16, 17]. MoM bearings are made of highly polished cobalt-chromium-molybdenum alloy leading to elevated Co and Cr in blood, urine, and hair of patients [18].

The potential long term side effects of MoM bearings wear products have always been a concern to orthopaedic community and significant efforts have been made in the first decade of 21st century to identify a suitable and reliable method to monitor the in vivo wear rate and identify failing implants [8, 10, 19–22]. Various blood fractions including serum, plasma, whole blood, and erythrocytes have been used in past studies to study Co and Cr levels in patients with MoM hip implants [18, 20, 22–24]. There is no consensus in the orthopaedic community about the most suitable blood fragment due to paucity of comparative evidence. This is further confounded by inaccuracies in reporting of the blood fractions used for analysis which was highlighted in our previously published work. The other potential reason for this is heterogeneity of consumables used in the studies and actual laboratory analysis techniques [25].

In 2007, Daniel et al. assessed the validity of serum levels of Co and Cr as a surrogate measure of systemic exposure to metal ions in hip replacement in 262 specimens [10]. They compared Co and Cr levels in paired samples of whole blood and serum in heterogenous cohort of patients with hip resurfacings and MoM THRs. They concluded that these metal ions levels in both blood fragments cannot be used either interchangeably or interconvertibly. They also concluded that difference in both fractions was concentration dependent. Our results also show similar pattern of Co and Cr concentrations in whole blood and plasma.

In 2008, Walter et al. compared the distribution of Cr and Co ions in whole blood, plasma, serum, and erythrocytes after 54 mm size Birmingham hip resurfacings in 29 patients (Smith & Nephew, United Kingdom) [22]. They concluded that the majority of Cr and Co were found in extracellular space in blood. Serum and plasma had the highest concentration with the least concentration being in the erythrocytes. The levels in whole blood were in between these two blood fractions. More importantly, serum and plasma levels mirror each other. They recommended the use of serum or plasma fractions for Co and Cr levels. In the same year, De Smet et al. published their work about the use of metal ion measurement as a diagnostic tool to identify problems with MoM hip resurfacing. They stated that serum Cr > 17 *μ*g/L and Co > 19 *μ*g/L are associated with metallosis [8]. Again, there were some inaccuracies in reporting of blood fraction as the samples were collected in anticoagulated containers which were centrifuged. This means the levels were measured in “plasma” and not in “serum” as reported. Based on this evidence we decided to use “plasma” as suitable blood fraction to measure metal ions and the Ghent University Laboratory became our external quality assurance partner.

In April 2010 the MHRA published its first guidance on MoM hips. This recommended using whole blood to measure Co and Cr levels and that a level of more than 7 ppb is a cause of concern [6]. Since we already had a laboratory system in place to measure metal ions in plasma rather than whole blood, it was important for us know the interchangeability between them. The other aim was to identify and if possible to derive a reliable and constant conversion ratio for Co and Cr levels in plasma and whole blood. Vendittoli et al. suggested the conversion ratio to be 1.39 and 1.37 for Co and Cr, respectively, in 64 patients with Durom hip arthroplasty (Zimmer, Winterthur, Switzerland) [21]. There was no evidence available regarding relationship and conversion ratio between metal ions in plasma and whole blood. The results of our study from this heterogeneous group show that the Co and Cr levels in plasma and whole blood cannot be used interchangeably. More importantly, the ratio of Co and Cr between plasma and whole blood is concentration dependent and it is not possible to derive a constant conversion ratio.

There are some limitations of this study. This cohort consists of patients with different implant brands and sizes of hip resurfacings and THRs with possibly different wear characteristics. We have not excluded any patients affected by possible factors influencing concentrations of Co and Cr but we assume that it should not affect the actual relationship of metal ions levels in plasma and whole blood [21, 26]. The metal ions analysis has been performed at a single laboratory and it is possible to encounter unexpected analysis malfunction but our laboratory process regularly undergoes external quality assurance (EQA) and we have not encountered any issues to date.

It is important to understand that metal ions concentrations in MoM hips patients are an adjunct in the management of these patients for the investigations of possible ARMD. Malek et al. recommended that future management strategy should not be solely based on metal ions level due to its poor sensitivity and specificity and predictor values [27]. We feel that it is more important to assess the trend of metal ions levels in sequential samples and this trend should be evident regardless if the metal ions were measured in either plasma or whole blood. As plasma Co and Cr levels are generally higher than whole blood, the same cut-off level of >7 *μ*g/L may lead to overinvestigation of patients with potential ARMD.

More recently the US Food and Drug Administration has issued advice about the investigation of MoM bearings and recommended measurement of Co levels in EDTA containing whole blood in symptomatic patients only. It does not recommend routine metal ions testing in asymptomatic patients [28]. This study confirms that plasma and whole blood Co and Cr levels cannot be used interchangeably or interconvertibly. We recommend the investigating clinician to be aware of process used at their metal ions analysis laboratory and not to mix and match the results from previously published studies.

## Figures and Tables

**Table 1:** Patients and procedures demographics excluding six patients with metal ions below detection limit (THR: Total Hip Replacement).

Demographics	
Age (range)	65.7 years (31–88 years)
Sex (M : F)	47 : 73
Mean implantation time (range)	59.3 months (10–173 months)
Procedure	THR: 113, resurfacing: 7
Laterality	Unilateral: 102, bilateral: 18
